# Minimally Invasive transCervical oEsophagectomy (MICE) for oesophageal cancer: prospective cohort study (IDEAL stage 2A)

**DOI:** 10.1093/bjs/znae160

**Published:** 2024-07-10

**Authors:** Richard J M T Vercoulen, Linde van Veenendaal, Irene F Kramer, Merlijn Hutteman, Atsushi Shiozaki, Hitoshi Fujiwara, Camiel Rosman, Bastiaan R Klarenbeek

**Affiliations:** Department of Surgery, Radboud University Medical Centre, Nijmegen, The Netherlands; Department of Surgery, Radboud University Medical Centre, Nijmegen, The Netherlands; Department of Surgery, Radboud University Medical Centre, Nijmegen, The Netherlands; Department of Surgery, Radboud University Medical Centre, Nijmegen, The Netherlands; Department of Surgery, Kyoto Prefectural University of Medicine Hospital, Kyoto, Japan; Department of Surgery, Kyoto Prefectural University of Medicine Hospital, Kyoto, Japan; Department of Surgery, Radboud University Medical Centre, Nijmegen, The Netherlands; Department of Surgery, Radboud University Medical Centre, Nijmegen, The Netherlands

## Abstract

**Background:**

Minimally invasive transcervical oesophagectomy is a surgical technique that offers radical oesophagectomy without the need for transthoracic access. The aim of this study was to evaluate the safety and feasibility of the minimally invasive transcervical oesophagectomy procedure and to report the refinement of this technique in a Western cohort.

**Methods:**

A single-centre prospective cohort study was designed as an IDEAL stage 2A study. Patients with oesophageal cancer (cT1b–4a N0–3 M0) who were scheduled for oesophagectomy with curative intent were eligible for inclusion in the study. The main outcome parameter was the postoperative pulmonary complication rate and the secondary outcomes were the anastomotic leakage, recurrent laryngeal nerve palsy, and R0 resection rates, as well as the lymph node yield.

**Results:**

In total, 75 patients underwent minimally invasive transcervical oesophagectomy between January 2021 and November 2023. Several modifications to the surgical technique were registered, evaluated, and implemented in the context of IDEAL stage 2A. A total of 12 patients (16%) had postoperative pulmonary complications, including pneumonia (4 patients) and pleural effusion with drainage or aspiration (8 patients). Recurrent laryngeal nerve palsy was observed in 33 of 75 patients (44%), with recovery in 30 of 33 patients (91%). A total of 5 of 75 patients (7%) had anastomotic leakage. The median number of resected lymph nodes was 29 (interquartile range 22–37) and the R0 resection rate was 96% (72 patients).

**Conclusion:**

Introducing minimally invasive transcervical oesophagectomy for oesophageal cancer in a Dutch institution is associated with a low rate of postoperative pulmonary complications and a high rate of temporary recurrent laryngeal nerve palsy.

## Teaser

Oesophageal cancer patients in Asia have been treated with the minimally invasive transcervical oesophagectomy procedure for a few years, showing promising results. The transcervical approach offers an oncologically radical resection without the need for transthoracic access to the mediastinum, resulting in less postoperative pulmonary complications. These outcomes are confirmed by this first series of 75 patients in a Western setting, laying the foundation for further development and implementation.

## Introduction

Radical oesophagectomy with two-field lymphadenectomy is part of the multimodal curative treatment of oesophageal cancer. Transthoracic minimally invasive oesophagectomy^1^ reduces postoperative morbidity compared with open surgery, but the complication rates are still high. Pulmonary and anastomotic complications are seen in up to 35% and 20% of patients respectively^2^. Transhiatal oesophagectomy (open or minimally invasive) is an alternative approach and this technique is associated with a lower pulmonary complication rate^3–5^. As only the distal mediastinal lymph nodes can be removed, the oncological safety is under debate, despite studies showing similar survival compared with transthoracic oesophagectomy^6–9^.

Minimally Invasive transCervical oEsophagectomy (MICE) is a novel procedure, in which the oesophagus is dissected *en bloc* with thoracic lymph nodes via single-port mediastinoscopy through a neck incision. The lower oesophagus, stomach, and abdominal lymph nodes are dissected by laparoscopy. MICE combines the advantages of transhiatal and transthoracic approaches and, as such, may achieve optimal oncological radicality and a low risk of pulmonary complications.

Several case series on MICE have been published from Japanese and Chinese centres, showing favourable postoperative complication rates and good oncological outcomes^10–13^. MICE has not been performed within hospitals outside of Asia, given the complexity of the surgical technique, the differences between Asian and Western patients, and the possible lack of prospective data showing its benefit over transthoracic oesophagectomy. Recently, it was shown that MICE can be safely introduced into clinical practice according to the IDEAL framework^14^. The aim of the present study was to report the safety and feasibility of MICE in a Dutch tertiary referral hospital according to the IDEAL 2A recommendations.

## Methods

### Patients

This study was designed as an IDEAL stage 2A single-centre study. Patients with resectable oesophageal cancer (cT1b-4a N0–3 M0) were eligible for MICE. During the study interval, the indication for MICE varied from proximal to mid oesophageal tumours and/or suspected upper mediastinal lymph node metastases to all oesophageal tumours (including distal tumour localization). According to Dutch national guidelines, all patients were offered five weekly cycles of carboplatin and paclitaxel with concurrent radiotherapy (41.4 Gy in 23 fractions, 5 days per week) followed by surgery according to the chemoradiotherapy for oesophageal cancer followed by surgery study (CROSS)^15,16^. After neoadjuvant chemoradiotherapy, patients were restaged using PET-CT to exclude distant metastases, were re-evaluated for surgery, and underwent MICE after oral and written informed consent.

### Definitions

Patients were graded according to the ASA risk classification before surgery^17^. Postoperative complications were categorized and described following the Clavien–Dindo Classification of Surgical Complications and the Esophagectomy Complications Consensus Group (‘ECCG’) classification^18–20^. Postoperative pulmonary complications were defined according to the Dutch Upper Gastrointestinal Cancer Audit (DUCA), that is pneumonia, pleural effusion including drainage or aspiration, thorax empyema, acute respiratory distress syndrome (‘ARDS’), or reintubation. Pneumonia was defined and classified in accordance with the Uniform Pneumonia Score^21^. When patients displayed signs of postoperative hoarseness, a laryngoscopy was conducted by an ear/nose/throat specialist on postoperative day 7. Recurrent laryngeal nerve palsy was confirmed when the laryngoscopy showed unilateral or bilateral vocal cord palsy. Persistent recurrent laryngeal nerve palsy was defined as a minimum of 1 year of vocal cord palsy, confirmed by laryngoscopy, as described above. Tumour staging was based on the UICC TNM Classification of Malignant Tumours (8th Edition)^22^ and lymph node stations were classified according to the Japanese Classification of Esophageal Cancer (11th Edition)^23,24^.

### Minimally invasive transcervical oesophagectomy

A central left collar incision was made, followed by lateralization of the strap muscles and exposure of the thyroid and trachea. After dissection of the left cervical para-oesophageal lymph nodes (101L), the left recurrent laryngeal nerve was identified, tested with an intraoperative nerve monitoring system (NIM 3.0, Medtronic, Minneapolis, MN, USA), and spared. After mobilizing the cervical oesophagus, a single-port platform (GelPoint Mini, Applied Medical, Rancho Santa Margarita, CA, USA) was placed, and three trocars were inserted. After creating a pneumomediastinum (8 mmHg), sharp dissection opened the plane outside the meso-oesophagus; downward development of this plane was facilitated by pneumodissection in the confined space of the mediastinum, enabling radical *en bloc* dissection of the upper and mid oesophagus and its lymph nodes, while exposing and preserving, both recurrent laryngeal nerves, the thoracic duct, the azygos vein, both pleurae, and the proximal part of both vagal nerves beyond the inferior borders of both main bronchi. During the transcervical phase, the following lymph node stations were dissected: bilateral paraoesophageal nerves, 105, 108, and 110; bilateral paratracheal nerves, 106, 106tbR, and 106tbL; subcarinal nerves, 107; and bilateral recurrent laryngeal nerves, 106recL and 106recR. Proximal (that is cervical) lymph nodes alongside both recurrent laryngeal nerves (101L, 106tbL, 101R, and 106tbR) could be approached directly through the same left collar incision. Below the level of the thoracic inlet, the lymph node dissection was performed using a single-port platform (GelPoint Mini, Applied Medical, Rancho Santa Margarita, CA, USA); right recurrent laryngeal nerve lymph nodes (106recR) were dissected from the retrotracheal plane.

A rendezvous between the transcervical dissection plane and the mediastinal space was achieved using a laparoscopic transhiatal approach. After mobilization of the greater and lesser curvature of the stomach and abdominal lymph node dissection (including stations 1, 2, 3, 7, 8a, 8p, 9, and 11), a gastric conduit was formed. Under direct laparoscopic view, the gastric conduit was brought up to the cervical incision for a handsewn end-to-side anastomosis. Fujiwara *et al*.^10,25,26^ have reported a more detailed description of the surgical technique.

### IDEAL framework

The IDEAL framework describes the stages of surgical innovation: Idea, Development, Exploration, Assessment, and Long-term follow-up. The introduction process of MICE in the Netherlands is in IDEAL stage 2A, in which the development of the procedure is the main goal. Besides the surgical outcomes of this safety and feasibility study, refinement of the surgical technique was registered to reach a state of sufficient stability to allow replication by others. To monitor adjustments to the surgical procedure, an evaluation form was developed to assess each MICE procedure. A total of four different phases were identified: cervical, mediastinal, abdominal, and anastomosis. For each phase, specific steps, such as lymphadenectomy or identification of the recurrent laryngeal nerve, could be assessed with regard to the level of contentment. Comments, details, changes, and areas of improvement for the next procedure were recorded. This form was available online, as well as on a desktop computer and on a mobile device to facilitate accessibility (Microsoft 365, Forms).

### Statistics

Data from numerical and categorical variables are summarized using the median (interquartile range (i.q.r.)) and the number of patients (%) respectively. The 2-year overall survival was calculated as the percentage of patients alive at 2-year follow-up. All statistical analyses were performed using SPSS^®^ (IBM, Armonk, NY, USA; Statistics for Windows, Version 27.0).

### Ethics

The introduction of MICE at Radboud University Medical Centre was concordant with the Dutch guideline ‘New Interventions into Clinical Practice’. This study does not fall within the scope of the Dutch Medical Research Involving Human Subjects Act. An official exemption from the local Medical Ethics Committee ‘Commissie Mensgebonden Onderzoek’ Arnhem-Nijmegen and participating hospitals was obtained. This study is reported according to the STROBE guidelines^27^.

## Results

A total of 75 patients operated on between January 2021 and November 2023 were included. The patient characteristics are shown in *Table 1*. A total of 73 of 75 patients (97%) underwent neoadjuvant chemoradiotherapy. CROSS was not indicated in two patients, because of early-stage oesophageal carcinomas in one patient with Barrett’s oesophagus and because of a stenotic oesophagus after caustic ingestion in the other patient (more than 40 years ago).

**Table 1:** Patient characteristics (*n* = 75)

Characteristics	Values
Age (years), median (interquartile range)	70 (64–74)
**Sex**	
Male	56 (75)
Female	19 (25)
**ASA grade**	
I	2 (3)
II	51 (68)
III	22 (29)
**Tumour location**	
Proximal–mid oesophagus	15 (20)
Distal oesophagus	60 (80)
**Histology**	
Adenocarcinoma	58 (77)
Squamous cell carcinoma	15 (20)
Adenosquamous cell carcinoma	2 (3)
**cT**	
T1	1 (1.3)
T2	10 (13.3)
T3	52 (69.3)
T4	1 (1.3)
Missing	11 (14.7)
**cN**	
N0	17 (22.7)
N1	23 (30.7)
N2	20 (26.7)
N3	4 (5.3)
Missing	11 (14.7)
**cM**	
M0	61 (81.3)
Mx	14 (18.7)
**Neoadjuvant therapy**	
Chemoradiotherapy	73 (97.3)
No therapy	2 (2.6)

Values are *n* (%) unless otherwise indicated.

Perioperative outcomes are shown in *Table 2*. Postoperative pulmonary complications were observed in 12 of 75 patients (16%). This included four patients with pneumoniae (Clavien–Dindo grade II) and eight patients with pleural effusion with the need for drainage or aspiration (Clavien–Dindo grade IIIa).

**Table 2:** Perioperative outcomes (*n* = 75)

Outcomes	Values
**Peroperative outcomes**	
Operation time (min), median (interquartile range)	360 (311–390)
Blood loss (ml), median (interquartile range)	100 (50–200)
Conversion	7 (9)
**Postoperative outcomes**	
Pulmonary complications	12 (16)
Pneumonia	4 (5)
Pleural effusion with drainage or aspiration	8 (11)
Anastomotic leakage	5 (7)
Clavien–Dindo grade I	3/5 (60)
Clavien–Dindo grade IIIb	2/5 (40)
Postoperative hoarseness	36 (48)
Confirmed recurrent laryngeal nerve palsy	33 (44)
Vocal cord augmentation	14/33 (42)
Spontaneous recovery	30/33 (91)
Duration of ICU/PACU stay (days), median (interquartile range)	1 (1–2)
Duration of postoperative hospital stay (days), median (interquartile range)	8.5 (7–11)
Thirty-day mortality	0 (0)
Ninety-day mortality	2 (3)
**Pathology**	
Lymph node yield, median (interquartile range)	29 (22–37)
Patients with tumou**r**-positive lymph nodes	43 (57)
Positive lymph node yield, median (interquartile range)	3 (1–4)
R0 resection	72 (96)
pT	
T0	16 (21)
T1	17 (23)
T2	7 (9)
T3	34 (45)
T4	1 (1
pN	
N0	30 (40)
N1	19 (25)
N2	21 (28)
N3	5 (7)
pM	
M0	70 (93)
M1	1 (1)
Mx	4 (5)
**Two-year overall survival, *n* = 30**	18 (60)

Values are *n* (%) unless otherwise indicated. ICU, intensive care unit; PACU, post anesthesia care unit.

A total of 9 of 75 MICE procedures (12%) were converted into transthoracic minimally invasive oesophagectomy procedures, due to suspicion of a tumour invading adjacent organs (pericardium or airway), loss of surgical view in the mediastinum because of obesity, or a thoracic inlet too narrow for access to the mediastinum. Anastomotic leakage was observed in 5 of 75 patients (7%), including 2 patients who underwent surgical re-intervention (Clavien–Dindo grade IIIb).

Postoperative hoarseness was present in 36 of 75 patients (48%). In 33 of 75 (44%) patients recurrent laryngeal nerve palsy was confirmed by postoperative laryngoscopy and in 14 patients vocal cord augmentation was performed. None of these 14 patients had relapse of hoarseness at 1-year follow-up. A total of 30 of 33 patients (91%) showed full recovery from recurrent laryngeal nerve palsy 1 year after surgery.

The surgical margin was tumour free (pR0) in 72 patients (96%). The median lymph node yield per patient was 29 (i.q.r. 22–37). *Fig. S1* shows an intraoperative snapshot after subcarinal lymph node dissection. During the study interval, 30 patients completed 2-year follow-up and 18 were alive at 2 years (giving a 2-year overall survival rate of 60%).

Between February 2022 and September 2023, 18 evaluation forms for MICE were completed. This led to changes in surgical technique, which are shown in detail in the *Supplementary Results*. Briefly, these changes included: use of recurrent laryngeal nerve monitoring; inclusion of distal oesophageal tumours; preservation of the strap muscles; a different order of procedural steps (mediastinal or abdominal first; dorsal or ventral mobilization of the upper mediastinal oesophagus first); use of different camera systems (5 mm flexible tip, three-dimensional, or 10 mm 30°); exploring different extraction sites (neck or abdomen); a lower threshold for conversion in case of a difficult cervical approach; development of a total laparoscopic pull-through procedure for the gastric tube; and changes in the surgical staff. Most recently, a new approach to the left recurrent laryngeal nerve was implemented, preserving its attachments to the lateral vascular sheet for better vascularization and protection (see *Fig. S2*).

## Discussion

This study shows that MICE after neoadjuvant chemoradiotherapy is associated with low rates of postoperative pulmonary complications and acceptable pathological outcomes. These findings support the careful adoption of MICE, which offers radical oesophagectomy with two-field lymphadenectomy without the need for transthoracic access. The main drawback of the procedure is a high rate of recurrent laryngeal nerve palsy, although 91% of patients recovered^28^. With progression through the learning curve and the use of tools like intraoperative nerve monitoring, recurrent laryngeal nerve palsy rates might be reduced^29^. Recurrent laryngeal nerve palsy in the present study did not seem to lead to additional morbidity (that is pneumonia) and vocal cord augmentation was of value to reduce hoarseness, as a bridge to spontaneous recovery^30^.

When comparing the outcome of this study with the outcomes of the Dutch Upper GI Cancer Audit data set or Japanese and Chinese studies, the results of the present study are encouraging^10–13^. An anastomotic leakage rate of 7% is low, even compared with transthoracic oesophagectomy, considering the fact that a cervical anastomosis is usually associated with a higher risk of anastomotic leakage^31^. Optimal exposure in the neck after removal of the cervical lymph nodes, a more distal location of the anastomosis, and increased experience with the cervical anastomosis might have contributed to this result. Moreover, most leakages could be treated by opening of the cervical wound or laparoscopic transhiatal drainage. The less severe sequelae of anastomotic leakage after MICE might be explained by preservation of the pleurae limiting the development of pleural empyema.

MICE can be considered radical oesophagectomy with two-field lymphadenectomy. This was confirmed by an acceptable lymph node yield and R0 resection rate. However, future studies will have to assess and determine the number of resected lymph nodes per nodal station and its anatomical borders. The 2-year overall survival rate of the first 30 patients was 60%, which is in line with data from the National Cancer Registry. Analyses of recurrence rates, recurrence free survival, and overall survival will be performed when the cohort reaches sufficient follow-up.

This study is an IDEAL stage 2A study and was therefore performed during the learning curve. Studies have shown that the learning curve for transthoracic minimally invasive oesophagectomy involves 119 consecutive cases before reaching the plateau phase with regard to postoperative complications^32,33^. Hence, outcomes of MICE may become even better with increasing experience of the surgical team, as only 75 patients have been operated on so far. Another important aspect of the current stage of development within IDEAL stage 2A^34^ is the adjustment of the surgical technique. The refinements made during the study are important, as they make MICE more suitable for the patient population, surgical team, and equipment, and will eventually contribute to reaching the plateau phase of the learning curve.

When introducing a new complex surgical procedure, such as MICE, collaboration with peers in the same stage of innovation is key. There are some examples of unnecessary learning-associated morbidity when several pioneers started new procedures on an individual base^35,36^. An international collaborative group on the development of MICE has recently been established to align the surgical techniques and reach consensus on an optimal procedure. Within this group, important topics will be addressed, such as patient selection, handling of recurrent laryngeal nerve palsy, development of training programmes, and data collection for collaborative reporting of outcomes. This collaboration will be the basis for a comparative trial of MICE *versus* the standard procedure to assess a possible benefit of MICE in patients with oesophageal cancer within an IDEAL stage 3 design.

## Supplementary Material

znae160_Supplementary_Data

## Data Availability

Data will be made available on reasonable request.

## References

[1] van der Sluis PC, Schizas D, Liakakos T, van Hillegersberg R. Minimally invasive esophagectomy. Dig Surg 2020;37:93–100 31096214 10.1159/000497456

[2] Voeten DM, Busweiler LAD, van der Werf LR, Wijnhoven BPL, Verhoeven RHA, van Sandick JW et al Outcomes of esophagogastric cancer surgery during eight years of surgical auditing by the Dutch Upper Gastrointestinal Cancer Audit (DUCA). Ann Surg 2021;274:866–873 34334633 10.1097/SLA.0000000000005116

[3] Orringer MB, Sloan H. Esophagectomy without thoracotomy. J Thorac Cardiovasc Surg 1978;76:643–654 703369

[4] Davies AR, Sandhu H, Pillai A, Sinha P, Mattsson F, Forshaw MJ et al Surgical resection strategy and the influence of radicality on outcomes in oesophageal cancer. Br J Surg 2014;101:511–517 24615656 10.1002/bjs.9456

[5] Kauppila JH, Johar A, Gossage JA, Davies AR, Zylstra J, Lagergren J et al Health-related quality of life after open transhiatal and transthoracic oesophagectomy for cancer. Br J Surg 2018;105:230–236 29405281 10.1002/bjs.10745

[6] Omloo JM, Lagarde SM, Hulscher JB, Reitsma JB, Fockens P, van Dekken H et al Extended transthoracic resection compared with limited transhiatal resection for adenocarcinoma of the mid/distal esophagus: five-year survival of a randomized clinical trial. Ann Surg 2007;246:992–1000; discussion 1000–1001 18043101 10.1097/SLA.0b013e31815c4037

[7] Boshier PR, Anderson O, Hanna GB. Transthoracic versus transhiatal esophagectomy for the treatment of esophagogastric cancer: a meta-analysis. Ann Surg 2011;254:894–906 21785341 10.1097/SLA.0b013e3182263781

[8] Maas KW, Biere SS, Scheepers JJ, Gisbertz SS, van-der-Peet DL, Cuesta MA. Laparoscopic versus open transhiatal esophagectomy for distal and junction cancer. Rev Esp Enferm Dig 2012;104:197–202 22537368 10.4321/s1130-01082012000400005

[9] Mertens AC, Kalff MC, Eshuis WJ, Van Gulik TM, Van Berge Henegouwen MI, Gisbertz SS et al Transthoracic versus transhiatal esophagectomy for esophageal cancer: a nationwide propensity score-matched cohort analysis. Ann Surg Oncol 2021;28:175–183 32607607 10.1245/s10434-020-08760-8 PMC7752871

[10] Fujiwara H, Shiozaki A, Konishi H, Kosuga T, Komatsu S, Ichikawa D et al Perioperative outcomes of single-port mediastinoscope-assisted transhiatal esophagectomy for thoracic esophageal cancer. Dis Esophagus 2017;30:1–8 10.1093/dote/dox047 28859387

[11] Mori K, Yamagata Y, Aikou S, Nishida M, Kiyokawa T, Yagi K et al Short-term outcomes of robotic radical esophagectomy for esophageal cancer by a nontransthoracic approach compared with conventional transthoracic surgery. Dis Esophagus 2016;29:429–434 25809390 10.1111/dote.12345 PMC5132031

[12] Wang X, Li X, Cheng H, Zhang B, Zhong H, Wang R et al Single-port inflatable mediastinoscopy combined with laparoscopic-assisted small incision surgery for radical esophagectomy is an effective and safe treatment for esophageal cancer. J Gastrointest Surg 2019;23:1533–1540 30635830 10.1007/s11605-018-04069-w

[13] Tokairin Y, Nakajima Y, Kawada K, Hoshino A, Okada T, Ryotokuji T et al A feasibility study of mediastinoscopic radical esophagectomy for thoracic esophageal cancer from the viewpoint of the dissected mediastinal lymph nodes validated with thoracoscopic procedure: a prospective clinical trial. Esophagus 2019;16:214–219 30737707 10.1007/s10388-018-00656-7

[14] Klarenbeek BR, Fujiwara H, Scholte M, Rovers M, Shiozaki A, Rosman C. Introduction of Minimally Invasive transCervical oEsophagectomy (MICE) according to the IDEAL framework. Br J Surg 2023;110:1096–1099 36960594 10.1093/bjs/znad079 PMC10416700

[15] Eyck BM, van Lanschot JJB, Hulshof MCCM, van der Wilk BJ, Shapiro J, van Hagen P et al Ten-year outcome of neoadjuvant chemoradiotherapy plus surgery for esophageal cancer: the randomized controlled CROSS trial. J Clin Oncol 2021;39:1995–2004 33891478 10.1200/JCO.20.03614

[16] Shapiro J, van Lanschot JJB, Hulshof MCCM, van Hagen P, van Berge Henegouwen MI, Wijnhoven BPL et al Neoadjuvant chemoradiotherapy plus surgery versus surgery alone for oesophageal or junctional cancer (CROSS): long-term results of a randomised controlled trial. Lancet Oncol 2015;16:1090–1098 26254683 10.1016/S1470-2045(15)00040-6

[17] Hurwitz EE, Simon M, Vinta SR, Zehm CF, Shabot SM, Minhajuddin A et al Adding examples to the ASA-physical status classification improves correct assignment to patients. Anesthesiology 2017;126:614–622 28212203 10.1097/ALN.0000000000001541

[18] Dindo D, Demartines N, Clavien PA. Classification of surgical complications: a new proposal with evaluation in a cohort of 6336 patients and results of a survey. Ann Surg 2004;240:205–213 15273542 10.1097/01.sla.0000133083.54934.ae PMC1360123

[19] Low DE, Alderson D, Cecconello I, Chang AC, Darling GE, D'Journo XB et al International consensus on standardization of data collection for complications associated with esophagectomy: Esophagectomy Complications Consensus Group (ECCG). Ann Surg 2015;262:286–294 25607756 10.1097/SLA.0000000000001098

[20] Zaninotto G, Low DE. Complications after esophagectomy: it is time to speak the same language. Dis Esophagus 2016;29:580–582 26043784 10.1111/dote.12375

[21] Seesing MFJ, Wirsching A, van Rossum PSN, Weijs TJ, Ruurda JP, van Hillegersberg R et al Defining pneumonia after esophagectomy for cancer: validation of the uniform pneumonia score in a high volume center in North America. Dis Esophagus 2018;31:doy002 10.1093/dote/doy002 29668913

[22] Bertero L, Massa F, Metovic J, Zanetti R, Castellano I, Ricardi U et al Eighth Edition of the UICC Classification of Malignant Tumours: an overview of the changes in the pathological TNM classification criteria-what has changed and why? Virchows Arch 2018;472:519–531 29209757 10.1007/s00428-017-2276-y

[23] Japan Esophageal S . Japanese Classification of Esophageal Cancer, 11th Edition: part I. Esophagus 2017;14:1–36 28111535 10.1007/s10388-016-0551-7 PMC5222932

[24] Japan Esophageal S . Japanese Classification of Esophageal Cancer, 11th Edition: part II and III. Esophagus 2017;14:37–65 28111536 10.1007/s10388-016-0556-2 PMC5222925

[25] Fujiwara H, Shiozaki A, Konishi H, Kosuga T, Komatsu S, Ichikawa D et al Single-port mediastinoscopic lymphadenectomy along the left recurrent laryngeal nerve. Ann Thorac Surg 2015;100:1115–1117 26354650 10.1016/j.athoracsur.2015.03.122

[26] Fujiwara H, Shiozaki A, Konishi H, Otsuji E. Mediastinoscope and laparoscope-assisted esophagectomy. J Vis Surg 2016;2:125 29078513 10.21037/jovs.2016.07.08 PMC5638202

[27] von Elm E, Altman DG, Egger M, Pocock SJ, Gøtzsche PC, Vandenbroucke JP. The strengthening the reporting of observational studies in epidemiology (STROBE) statement. Epidemiology 2007;18:800–804 18049194 10.1097/EDE.0b013e3181577654

[28] Scholtemeijer MG, Seesing MFJ, Brenkman HJF, Janssen LM, van Hillegersberg R, Ruurda JP. Recurrent laryngeal nerve injury after esophagectomy for esophageal cancer: incidence, management, and impact on short- and long-term outcomes. J Thorac Dis 2017; 9(Suppl 8): S868–S878 28815085 10.21037/jtd.2017.06.92 PMC5538987

[29] Komatsu S, Konishi T, Matsubara D, Soga K, Shimomura K, Ikeda J et al Continuous recurrent laryngeal nerve monitoring during single-port mediastinoscopic radical esophagectomy for esophageal cancer. J Gastrointest Surg 2022;26:2444–2450 36221021 10.1007/s11605-022-05472-0

[30] Oshikiri T, Takiguchi G, Hasegawa H, Yamamoto M, Kanaji S, Yamashita K et al Postoperative recurrent laryngeal nerve palsy is associated with pneumonia in minimally invasive esophagectomy for esophageal cancer. Surg Endosc 2021;35:837–844 32086619 10.1007/s00464-020-07455-1

[31] van Workum F, Verstegen MHP, Klarenbeek BR, Bouwense SAW, van Berge Henegouwen MI, Daams F et al Intrathoracic vs cervical anastomosis after totally or hybrid minimally invasive esophagectomy for esophageal cancer: a randomized clinical trial. JAMA Surg 2021;156:601–610 33978698 10.1001/jamasurg.2021.1555 PMC8117060

[32] van Workum F, Stenstra MHBC, Berkelmans GHK, Slaman AE, Henegouwen MIV, Gisbertz SS et al Learning curve and associated morbidity of minimally invasive esophagectomy: a retrospective multicenter study. Ann Surg 2019;269:88–94 28857809 10.1097/SLA.0000000000002469

[33] Claassen L, Hannink G, Luyer MDP, Ainsworth AP, Henegouwen M, Cheong E et al Learning curves of Ivor Lewis totally minimally invasive esophagectomy by hospital and surgeon characteristics: a retrospective multinational cohort study. Ann Surg 2022;275:911–918 33605581 10.1097/SLA.0000000000004801

[34] McCulloch P, Altman DG, Campbell WB, Flum DR, Glasziou P, Marshall JC et al No surgical innovation without evaluation: the IDEAL recommendations. Lancet 2009;374:1105–1112 19782876 10.1016/S0140-6736(09)61116-8

[35] Wasmuth HH, Faerden AE, Myklebust TÅ, Pfeffer F, Norderval S, Riis R et al Transanal total mesorectal excision for rectal cancer has been suspended in Norway. Br J Surg 2020;107:121–130 31802481 10.1002/bjs.11459

[36] van Hilst J, de Rooij T, Bosscha K, Brinkman DJ, van Dieren S, Dijkgraaf MG et al Laparoscopic versus open pancreatoduodenectomy for pancreatic or periampullary tumours (LEOPARD-2): a multicentre, patient-blinded, randomised controlled phase 2/3 trial. Lancet Gastroenterol Hepatol 2019;4:199–207 30685489 10.1016/S2468-1253(19)30004-4

