# Supplementary material for: Minimally Invasive transCervical oEsophagectomy (MICE) for oesophageal cancer: prospective cohort study (IDEAL stage 2A)
Source: Br J Surg. 2024 Jul 10;111(7):znae160. doi: 10.1093/bjs/znae160 (PMC11235329; doi:10.1093/bjs/znae160)
Supplement: znae160_Supplementary_Data [file znae160_supplementary_data.docx]

**Supplementary files**

**Minimally invasive transcervical oesophagectomy (MICE) for oesophageal cancer: a prospective cohort study (IDEAL stage 2A)**

Richard J.M.T. Vercoulen MD^1^, Linde van Veenendaal MD PhD^1^, Irene F. Kramer MD PhD^1^, Merlijn Hutteman MD PhD^1^, Atsushi Shiozaki MD PhD^2^, Hitoshi Fujiwara MD PhD^2^, Camiel Rosman MD PhD^1^, Bastiaan R. Klarenbeek MD PhD^1^

**Affiliations**

^1^ Radboud University Medical Centre, Department of Surgery, Nijmegen, The Netherlands

^2^ Kyoto Prefectural University of Medicine Hospital, Department of Surgery, Kyoto, Japan

**Corresponding author**

Bastiaan R. Klarenbeek MD PhD

Radboud University Medical Centre

Department of Surgery

Geert Grooteplein 10 (route 618)

6500 HB Nijmegen, The Netherlands

Telephone: 0031 24 36 68086

eMail: bastiaan.klarenbeek@radboudumc.nl

**Supplementary Materials - Index**

| **Supplementary Methods** |  |
| --- | --- |
| None |  |
| **Supplementary Results** |  |
| IDEAL 2A modifications to the surgical procedure | Page 3 |
| **Supplementary Appendixes** |  |
| None |  |
| **Supplementary Figures** |  |
| Figure 1A and 1B  Figure 2A and 2B | Page 6  Page 7 |
| **References** |  |
| Supplementary references | Page 8 |

**Supplementary results**

**Supplementary files**

*IDEAL 2A modifications to the surgical procedure*

In the first ten procedures continuous nerve monitoring was used, but this did not help us in preventing neuropraxia of the recurrent laryngeal nerve. We did not find any correlation between duration of manipulation of the RLN and postoperative hoarseness or vocal cord paresis. The downside of continuous nerve monitoring is that it is distracting for the surgeon during the mediastinal dissection, especially in the beginning of the learning curve. We are considering reintroduction of continuous nerve monitoring, as we are further in our learning curve and sought for decreasing injury to the recurrent laryngeal nerve.

Patient selection for the MICE procedure changed. It was recognized that the patients with upper to middle oesophageal tumours or upper mediastinal lymph node metastasis has often advanced tumour stage and these were often difficult cases in the beginning of the learning curve. Some of these patients had a proximal squamous cell carcinoma that were also eligible for definitive chemoradiotherapy.

Together with our head and neck surgeons, the cervical dissection and port placement was changed. Instead of dividing the strap muscles, a medial approach is chosen where the the single-port platform behind medial to the strap muscles. This gives the same exposure of the RLN and oesophagus; and provides the same range of motion of the instruments for the mediastinal dissection.

In the first few patients it was found difficult to connect the transcervical dissection plane with the transhiatal plane. In some patients, the transhiatal dissection was done first. When the dissection of the mediastinum from the neck met with the transhiatal dissection plane, the pneumomediastinum was lost and your surgical field collapses. Therefore, the cervical dissection is done first. An important lesson from these changes is, that we can still do a laparoscopy first and check for peritoneal metastasis in patients with advanced tumours.

Different camera systems were tested for the transcervical mediastinal dissection. The 5mm flexible tip camera was found to be too sensitive for collision with other instruments in the small working space of the mediastinum. 3D technology was not considered an advantage in the small confined space of the mediastinum and the close proximity of the vital structures. The current camera system is a 10mm 30-degree angle EndoEye, with high resolution video (Olympus, Tokyo, Japan).

We had a low threshold for conversion from the beginning, especially since we had online instead of on-site proctoring by the Japanese expert surgeons. Still, conversion after a difficult mediastinal exploration could be a burden, because it entails the repositioning of patient from French to prone and back to French position for the pull-up of the gastric tube and cervical anastomosis, which is very time consuming. Therefore, with an early decision to convert during the cervical exploration, only one repositioning to prone is necessary after the abdominal dissection, to allow for our standard transthoracic procedure and intrathoracic anastomosis in prone.

We changed our procedure for gastric conduit formation and pull-through to the cervical incision. From an open gastric conduit formation and pull-trhough like we do in or regular McKeown cases, we now do a laparoscopic gastric conduit formation and pull-through under direct laparoscopic vision. Now only a very small abdominal extraction site is needed to take out the specimen. For this we use our cervical single-port platform at the end of the procedure after the cervical anastomosis and the cervical wound was closed.

Recently a new approach to the left recurrent laryngeal nerve was implemented preserving its attachments to the lateral vascular sheet for better vascularisation and protection of the nerve (see figure 3). This approach is not possible in all patients, since the position of the left recurrent laryngeal nerve is sometimes very medially in the tracheal-oesophageal groove. Lateralising the recurrent laryngeal nerve in these cases can possibly increase traction injury to the recurrent laryngeal nerve end-branches. The effects on recurrent laryngeal nerve palsy rates are yet to be evaluated.

There were changes in our surgical team. Every new member had to go through their own learning curve. The surgical view, anatomical orientation, handling of the camera and narrow space in the mediastinum were felt to be challenging, even for experienced upper GI surgeons.

**Supplementary Figures**

**
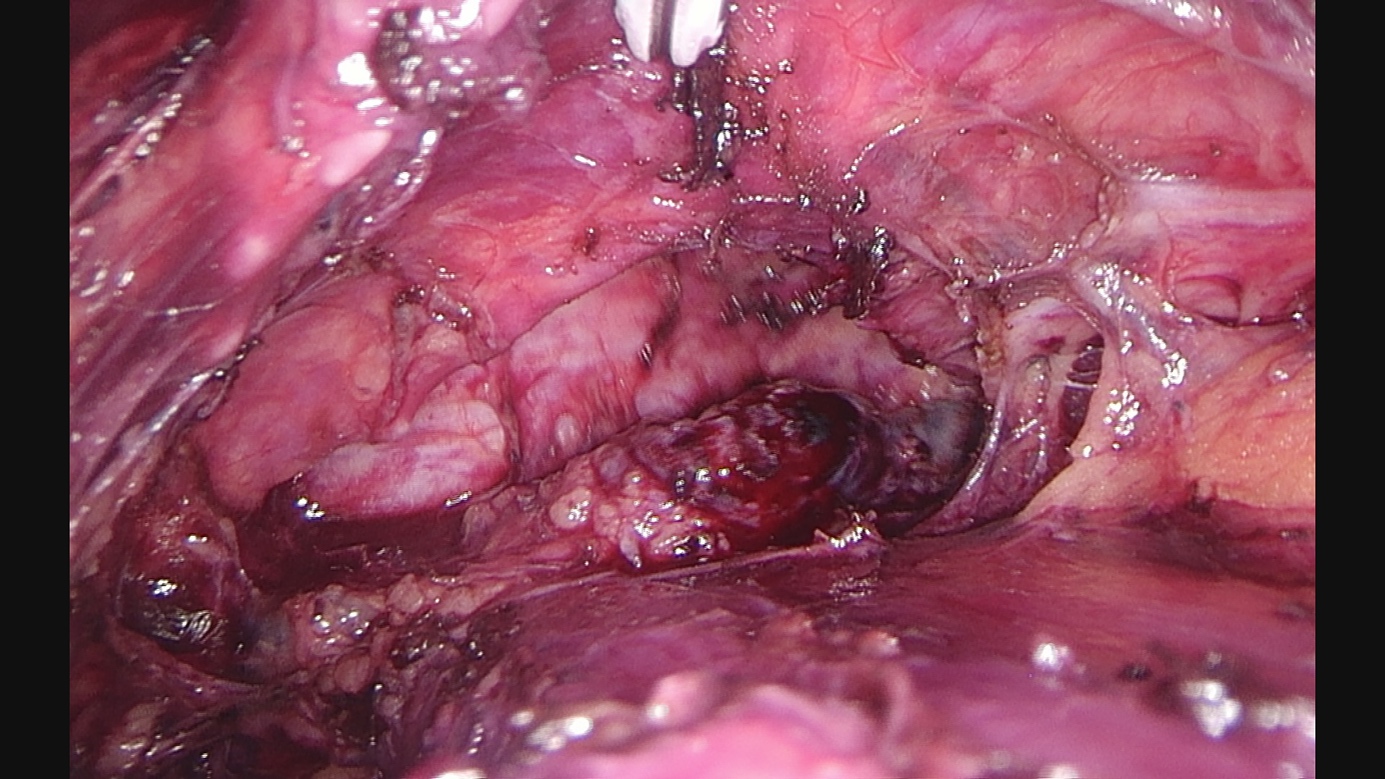
**

Figure S1A – image taken after the subcarinal lymph node dissection.


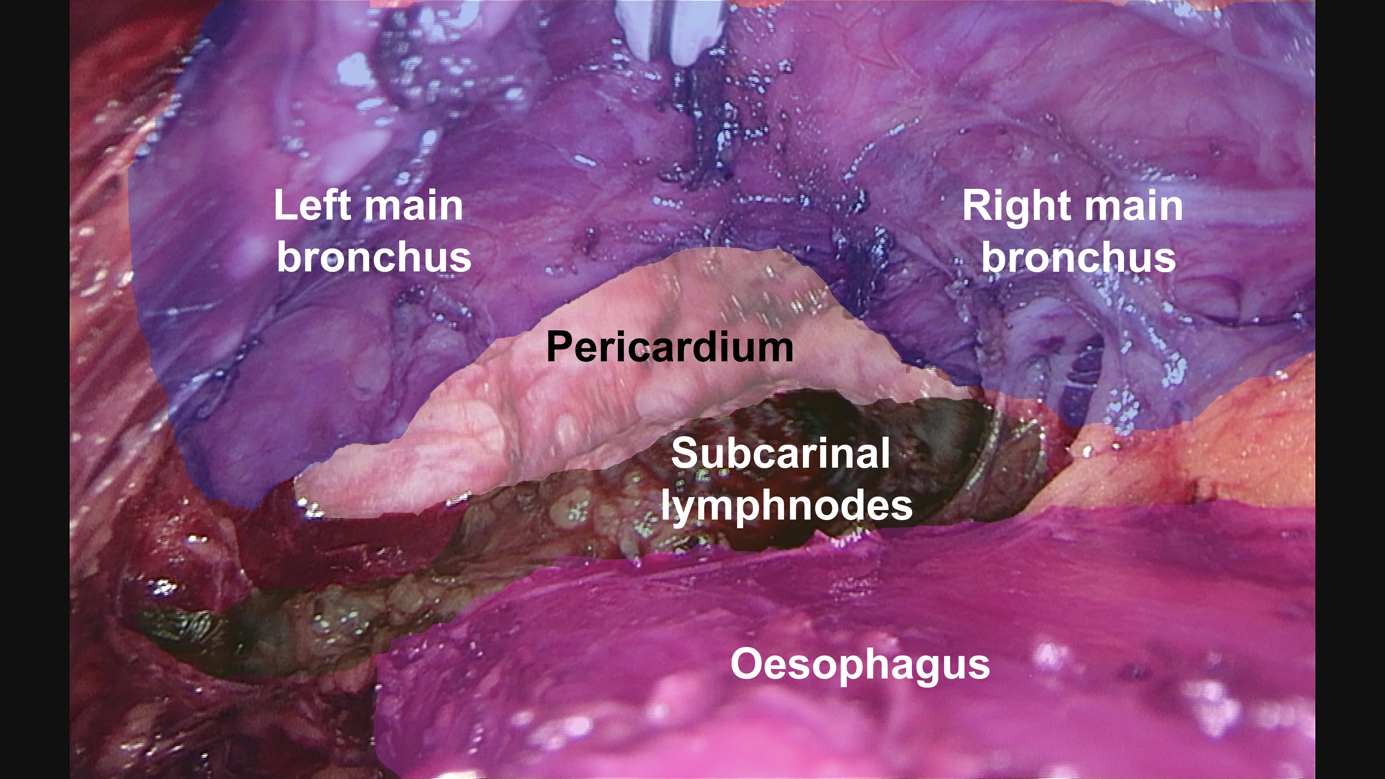


Figure S1B - anatomical structures depicted with overlays.


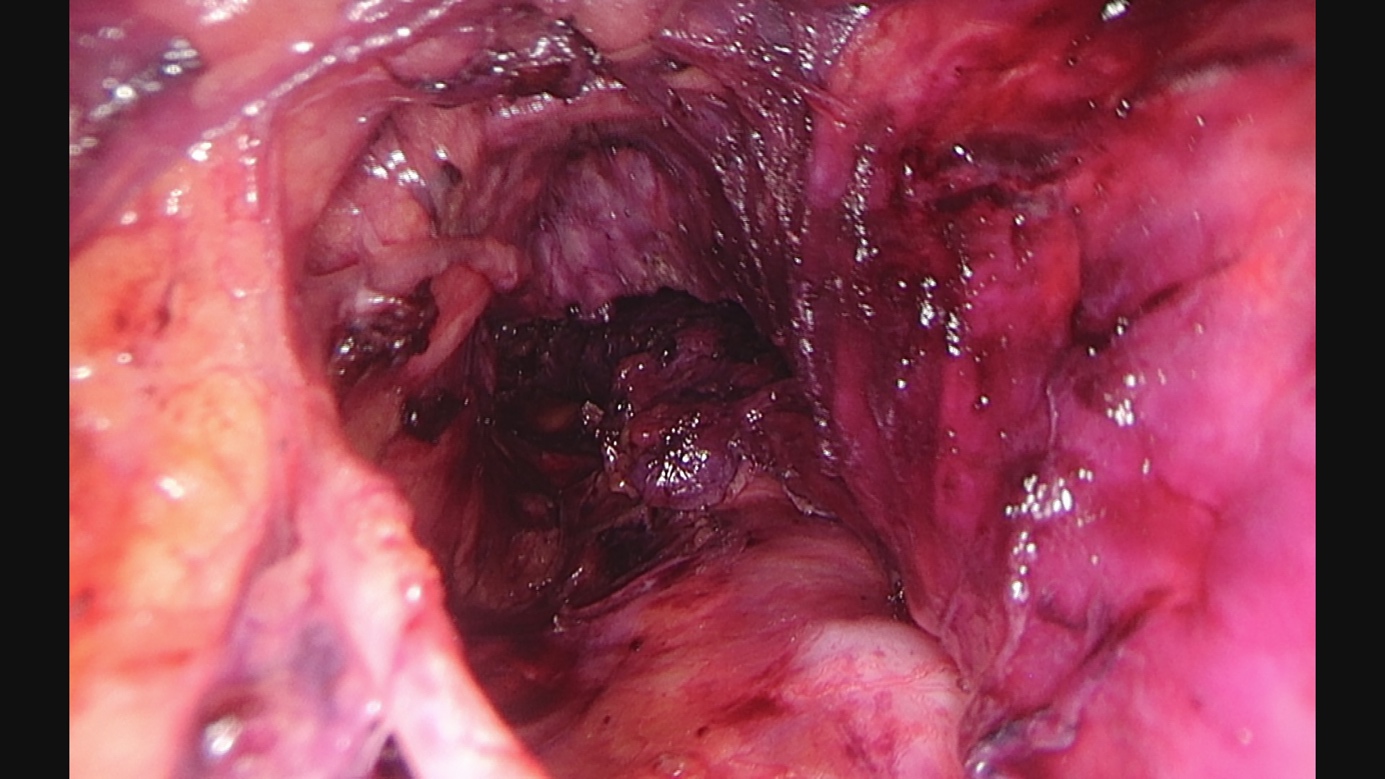


Figure S2A – image of dissection of left recurrent laryngeal nerve (an IDEAL 2A modification from the original MICE technique, as described by Fujiwara et al.^(25-27)^


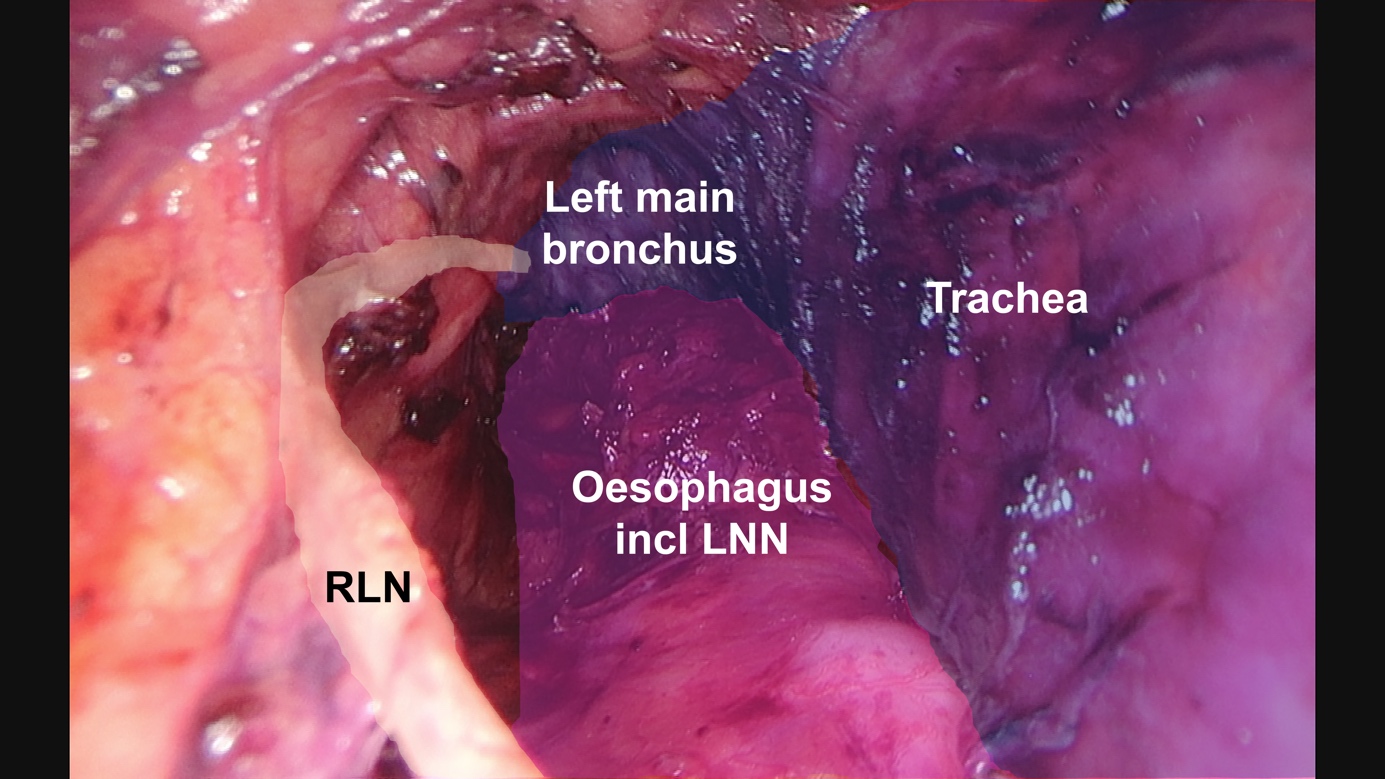


Figure S2B – anatomical structures depicted with overlays.

**Supplementary references**

1. Fujiwara H, Shiozaki A, Konishi H, Kosuga T, Komatsu S, Ichikawa D, et al. Perioperative outcomes of single-port mediastinoscope-assisted transhiatal esophagectomy for thoracic esophageal cancer. Diseases of the Esophagus. 2017;30:1-8
2. Fujiwara H, Shiozaki A, Konishi H, Kosuga T, Komatsu S, Ichikawa D, et al. Single-Port Mediastinoscopic Lymphadenectomy Along the Left Recurrent Laryngeal Nerve. Ann Thorac Surg. 2015;100(3):1115-1117
3. Fujiwara H, Shiozaki A, Konishi H, Otsuji E. Mediastinoscope and laparoscope-assisted esophagectomy. J Vis Surg. 2016;2:125.
